# Supplementary material for: Somatostatin, Cortistatin and Their Receptors Exert Antitumor Actions in Androgen-Independent Prostate Cancer Cells: Critical Role of Endogenous Cortistatin
Source: Int J Mol Sci. 2022 Oct 27;23(21):13003. doi: 10.3390/ijms232113003 (PMC9654089; doi:10.3390/ijms232113003)
Supplement: Supplementary file 1 [file ijms-23-13003-s001.zip › ijms-1908579-supplementary.pdf]

Supplemental Table S1: Specific primers for human transcripts used in this study.
NCBI accession number, primers sequences, and expected product sizes are included.

Gene	Accession Number	Primer Sequence (Sense)	Primer Sequence (Antisense)	Product Size (bp)
SST	NM_001048.4	AACCCAACCAGACGGAGAA	TAGCCGGGTTTGAGTTAGCA	111
CORT	NM_001302.5	CTCCAGTCAGCCCACAAGAT	CAAGCGAGGAAAGTCAGGAG	173
SSTR1	NM_001049	CACATTTCTCATGGGCTTCCT	ACAAACACCATCACCACCATC	165
SSTR2	NM_001050	GGCATGTTTGACTTTGTGGTG	GTCTCATTCAGCCGGGATTT	185
SSTR3	NM_001051	TGCCTTCTTTGGGCTCTACTT	ATCCTCCTCCTCAGTCTTCTCC	190
SSTR4	NM_001052	TCTTTGTGCTCTGCTGGATG	GGATAAGGGACACGTGGTTG	174
SSTR5	NM001053	CTGGTGTTTGCGGGATGTT	GAAGCTCTGGCGGAAGTTGT	183
SSTR5TMD4	DQ448304 (uniprot annotation)	TACCTGCAACCGTCTGCC	AGCCTGGGCCTTTCTCCT	98
SSTR5TMD5	D2CFK4 (uniprot annotation)	GCGCCGTCTTCATCATCTAC	CAGGAAAAGCTGGTGTTTGG	159
MKI67	NM_002417	GACATCCGTATCCAGCTTCCT	GCCGTACAGGCTCATCAATAAC	139
CDK2	NM_001798.4	GCTCTCACTGGCATTCCTCTT	GAGGTTTAAGGTCTCGGTGG	109
CDK4	NM_000075.3	ACAGTTCGTGAGGTGGCTTT	TACCTTGATCTCCCGGTCAG	111
CDK6	NM_001145306.2	TCGATGAACTAGGCAAAGACC	GTCCTGGAAGTATGGGTGAGA	101
CDKN1A	NM_000389.5	ATAAGGAAGCGACCTGCAAC	CACATGGTCTTCCTCTGCTGT	100
CDKN1B	NM_004064.5	ATAAGGAAGCGACCTGCAAC	TTGGGGAACCGTCTGAAA	88
CDKN2D	NM_001800.4	AACCGCTTCGGCAAGAC	GCTGGCACCTTGCTTCA	67
MMP3	NM_002422.5	ACTAGCAAGGACCTCGTTTTCA	AGGGTGTGGATGCCTCTTG	79
MMP9	NM_004994.2	CAGTGCCATGTAAATCCCCA	CACCTCCACTCCTCCCTTTC	102
CDH2	NM_001308176.2	AGACCGACCCAAACAGCA	GCAGCAACAGTAAGGACAAACA	91
EGF	NM_001178130.3	GCAATGTCCCTTTTTGGTGA	GTCCTTTCCAGTGTGTTTGTTG	87
EZH2	NM_004456.5	CACTCCTTTCATACGCTTTTCTG	TGTTTCTGTGTTCTTCCGCTTA	119
MYC	NM_001354870.1	CTCGGATTCTCTGCTCTCCTC	TTCCTCATCTTCTTGTTCCTCCT	124
PTEN	NM_000314.8	TAAAGCTGGAAAGGGACGAA	TGGTCCTTACTTCCCCATAGAA	110
VEGFR	NM_002253.3	TCTCTGCCTACCTCACCTGTTT	TGACTGATTCCTGCTGTGTTG	89
ACTB	NM_001101	ACTCTTCCAGCCTTCCTTCCT	CAGTGATCTCCTTCTGCATCCT	176
GADPH	NM_002046	AATCCCATCACCATCTTCCA	AAATGAGCCCCAGCCTTC	122
